# Impact of Proton Pump Inhibitors on Kidney Function and Chronic Kidney Disease Progression: A Systematic Review

**DOI:** 10.7759/cureus.49883

**Published:** 2023-12-03

**Authors:** Mihirkumar P Parmar, Safa Kaleem, Periyaiyadever Samuganathan, Lyluma Ishfaq, Tejawi Anne, Yashaswi Patel, Sashank Bollu, Roopeessh Vempati

**Affiliations:** 1 Internal Medicine, Gujarat Medical Education and Research Society, Vadnagar, IND; 2 Internal Medicine, Shadan Institute of Medical Sciences, Hyderabad, IND; 3 Internal Medicine, Manipal University College of Malaysia, Melaka, MYS; 4 Internal Medicine, Government Medical College Srinagar, Srinagar, IND; 5 Internal Medicine, Gandhi Medical College & Hospital, Secunderabad, IND; 6 Internal Medicine, Government Medical College Surat, Surat, IND; 7 Internal Medicine, Gandhi Medical College, Hyderabad, IND; 8 Internal Medicine, Gandhi Medical College & Hospital, Hyderabad, IND; 9 Cardiology, Heart and Vascular Institute, Detroit, USA

**Keywords:** long-term results, kidney injury, kidney function, chronic kidney disease, proton pump inhibitors

## Abstract

Proton pump inhibitors (PPIs) are widely prescribed medications for the management of various gastrointestinal disorders, primarily gastroesophageal reflux disease (GERD) and peptic ulcers. However, recent concerns have emerged regarding their potential adverse effects on kidney function and their role in the progression of chronic kidney disease (CKD). This systematic review aims to comprehensively analyze the existing literature to assess the impact of PPI use on kidney function and CKD progression. We took information from PubMed, PubMed Central (PMC), and Google Scholar articles from the last 10 years, from 2013 to 2023, and looked for links between PPI use and a number of kidney-related outcomes. These included acute kidney injury, a drop in the estimated glomerular filtration rate (eGFR), and new cases of CKD. The findings of this systematic review highlight the need for a thorough evaluation of the benefits and risks associated with PPI use, particularly in patients with pre-existing kidney conditions, in order to inform clinical decision-making and improve were taken out and looked at to see if there were any links between PPI use and different kidney-related events, such as acute kidney injury, a drop in the estimated eGFR, and the development of CKD. The review also explores potential mechanisms underlying PPI-induced nephrotoxicity. The findings of this systematic review highlight the need for a thorough evaluation of the benefits and risks associated with PPI use, particularly in patients with pre-existing kidney conditions, in order to inform clinical decision-making and improve patient care. Further research is warranted to better understand the complex interplay between PPIs, kidney function, and CKD progression.

## Introduction and background

This article was previously posted to the Research Square preprint server on September 21, 2023 [1].

Worldwide, proton pump inhibitors (PPIs) are extensively utilized in acid suppression therapy. In addition to antibiotics, they are frequently prescribed for a number of acid-related conditions, such as gastroesophageal reflux disease (GERD), peptic ulcer disease, esophagitis, gastritis, Barrett esophagus, and the removal of Helicobacter pylori. They are also frequently administered in conjunction with nonsteroidal anti-inflammatory medications (NSAIDs) and for preventive purposes.

An estimated 15 million people in the US are reported to be using prescription PPIs (estimated prevalence of 7.8% in the adult population) [2]. Because PPIs can also be purchased over-the-counter without a prescription, the actual prevalence of PPI use is probably substantially greater. Market research reports indicate that 385 million units of over-the-counter “heartburn” medications, of which PPIs account for 85% of the market, were sold in 2017, with an estimated $2.6 billion in sales [3]. Some studies show that people with chronic kidney disease (CKD) are given PPIs more often and for longer periods of time than people without CKD. This means that the number of people who use PPIs is probably higher in the CKD community than in the non-CKD community [4].

PPIs are frequently used for durations of usage and indications that the US Food and Drug Administration (FDA) has never evaluated or approved. They are commonly prescribed excessively, infrequently taken down, and frequently started incorrectly when a patient is in the hospital. Furthermore, their use is prolonged even when there is no medical need for it [5-10]. It is believed that between 53% and 69% of PPI prescriptions are written for unsuitable purposes [5,11], wherein in many situations the advantages of using PPIs (or not using them) may not outweigh the dangers [11-13].

## Review

Methods

**Figure 1:**
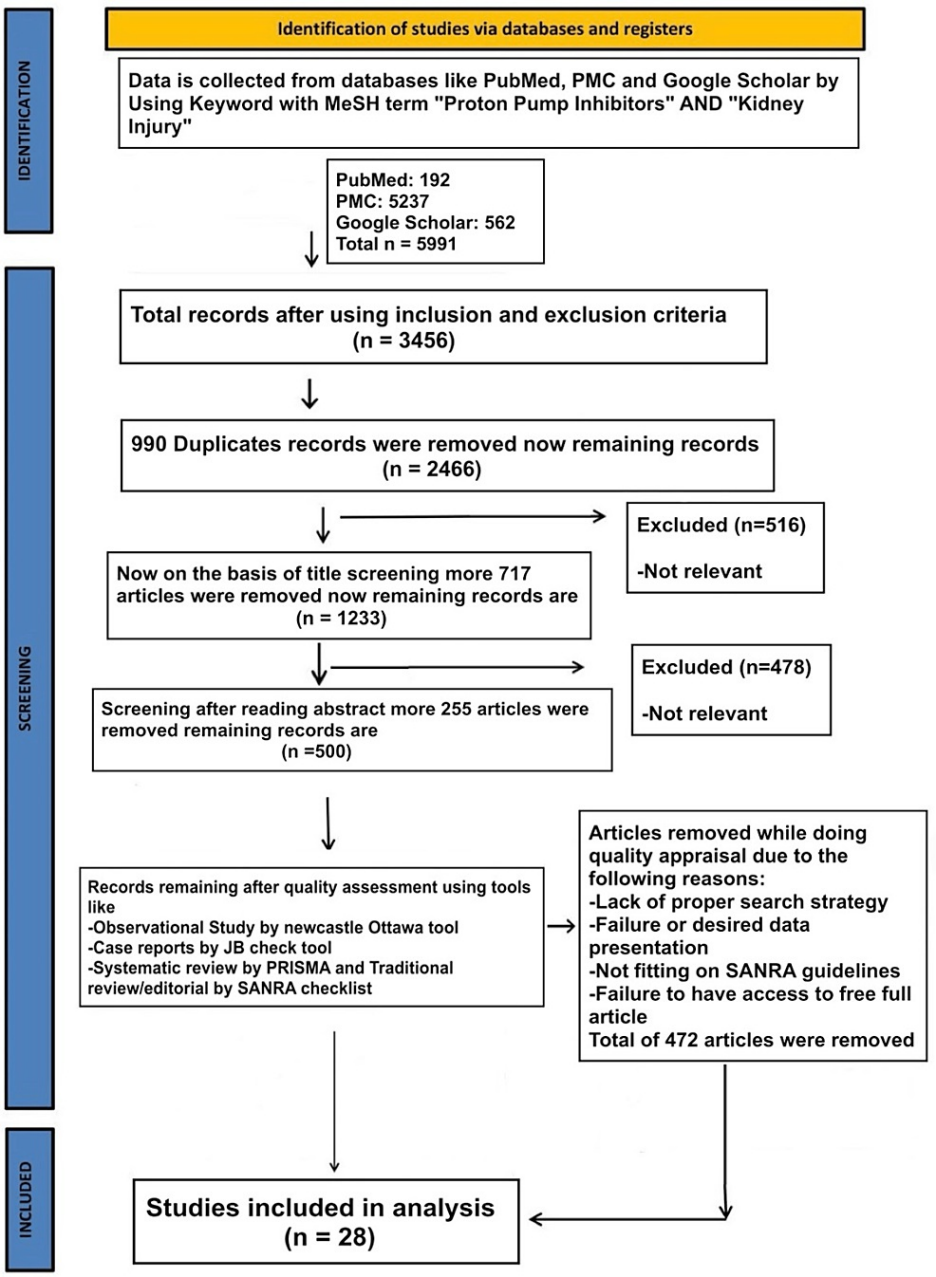
PRISMA flow chart PRISMA: Preferred Reporting Items for Systematic Review and Meta-Analysis; MeSH: Medical Subject Headings; SANRA: scale for quality assessment of narrative review articles; PMC: PubMed Central.

We conducted our systematic review in accordance with the Preferred Reporting Items for Systematic Reviews and Meta-Analysis (PRISMA) criteria (Figure 1). We used the following medical subject headings (MeSH) terms with keywords like “irritable bowel syndrome” and “kidney injury” to gather data from the National Library of Medicine (PubMed), PubMed Central (PMC), and Google Scholar. Articles from the last 10 years, 2013 to 2023, were gathered for this systematic review. Out of 192 PubMed, 5,237 PMC, 562, and 5,991 publications, 28 were included to create the result table.

Inclusion and Exclusion Criteria

In our analysis, we looked at all full-text papers, studies with people as subjects, and papers that were published in English. In the 10 years from 2013 to 2023, many studies were written about how PPIs affect kidney function and the progression of chronic kidney disease. These studies included clinical trials, controlled clinical trials, randomized controlled trials, observational studies, case-control studies, prospective and retrospective cohort studies, population-based cohort studies, cross-sectional studies, and observational studies. We did not include research that did not involve people or articles that did not have the full text.

Results

Results after combining all pre-existing selected studies are included here in Table 1, mentioning author, year of publication, WHO region, focus of study, findings, and key observations. The relevance and quality of the included studies are also assessed using the CASP checklist in Tables 2-5.

**Table 1:** Characteristics of studies proton pump inhibitors (PPIs), chronic kidney disease (CKD), gastroesophageal reflux disease (GERD), acute kidney injury (AKI), acute interstitial nephritis (AIN), nonsteroidal anti-inflammatory drugs (NSAIDs), Atherosclerosis Risk in Communities (ARIC), hospital-acquired acute kidney injury (HA-AKI), major adverse renal events (MARE), estimated glomerular filtration rate (eGFR), hazard ratio (HR), health maintenance organization (HMO), odd ratio (OR), adjusted odd ratio (aOR), confidence interval (CI).

Citation and Year of Publication	WHO region	Country of the study	The focus of the study	Findings	Key observation
Hart, et al. 2019 [14]	Region of the Americas	United States of America	In a sizable population-based health maintenance organization (HMO) cohort, this retrospective cohort study investigated the relationship between PPI usage and the incident risk of AKI and CKD.	A significantly higher risk of AKI was linked to PPI exposure (adjusted odds ratio [aOR] 4.35, 95% confidence interval [CI] 3.14-6.04, p<0.0001). When compared to controls, PPIs were linked to a greater risk of chronic kidney disease (CKD) (aOR 1.20, 95% CI 1.12-1.28, p<0.0001).	An elevated incidence of incident AKI and CKD is linked to PPI use. Propensity score-matched analyses revealed that there were still relationships between PPI use and AKI and CKD.
Guedes, et al. 2020 [15]	Region of the Americas	Brazil	The objective of this retrospective cohort study was to examine the relationship between omeprazole use over time and the development of chronic kidney disease (CKD) in adults and older people.	Compared to non-users (10.5%), those taking omeprazole had a greater percentage of CKD development (70.6%). The hazard ratio of 7.34 (CI: 3.94-13.71) showed that omeprazole users had a higher chance of progressing to worse stages of CKD than non-users. Regarding the remaining characteristics, there was no discernible variation between the groups (p > 0.05).	Omeprazole use on a regular basis was significantly associated with worsening stages of chronic kidney disease (CKD) in adult and elderly patients.
Aurora, et al. 2016 [16]	Region of the Americas	United States of America	A prospective logistic regression analysis of the data was combined with two different retrospective case-control research designs to look at the link between taking PPIs and getting chronic kidney disease (CKD) or dying.	PPI-using patients had greater risks of developing CKD (OR 1.10, 95% CI 1.05–1.16) and dying (OR 1.76, 95% CI 1.67–1.84) than non-PPI-using patients, according to a prospective logistic analysis of case-control data.	Proton pump inhibitor use is linked to a higher chance of developing CKD and passing away. According to the study, using PPIs raised the likelihood of developing CKD by 10% and was linked to a 75% higher risk of death.
Lu, et al. 2022 [17]	Western Pacific Region	China	The purpose of the study was to determine if PPIs influence the metabolism of gut-derived uremic toxins, such as trimethylamine-N-oxide, p-cresyl sulfate, and indoxyl sulfate (IS), as a potential cause of chronic kidney disease (CKD).	The study found that when mice were given PPIs (omeprazole, lansoprazole, and pantoprazole at 30 mg/kg) for three weeks, only the IS plasma levels of the three gut-derived uremic toxins went up. PPIs increase the levels of CYP2E1 protein in the liver, which is an important enzyme in the creation of IS. This is because PPIs prevent this enzyme from being broken down, which raises the exposure to IS. It is noteworthy that kidney impairment with mild glomerular structural alterations and fibrosis symptoms only happened after three weeks of high-dose PPI treatment (30 mg/kg).	PPI use has been linked to an increased risk of chronic kidney disease (CKD). This association may be explained by the biological mechanism of PPI-induced IS production via increased hepatic CYP2E1 protein levels and increased IS exposure.
Shih-Chang, et al. 2018 [18]	East Asia	Republic of China (Taiwan)	This population-based case-control study aims to identify 16,704 cases of newly diagnosed CKD in individuals 20 years of age or older between 2000 and 2013.	Those who used PPIs had an OR for CKD of 1.41 (95% confidence interval (CI) 1.34, 1.48) when compared to those who had never used PPIs. Cumulative duration and dosage regression analysis shows a weak relationship between higher risks of CKD and almost all main forms of PPIs. The odds ratio (OR) for the total amount of time (month) spent using PPIs was 1.02 (95% CI 1.01, 1.02) and the OR for the total amount of time (microgram) spent using PPIs was 1.23 (95% CI 1.18, 1.28).	Taiwan health insurance claims data analysis using PPIs revealed 1.4-fold greater risks of CKD.
Rodríguez-Poncelas, et al. 2018 [19]	European region	Portugal (Southwest Europe)	People who were 15 years of age or older between January 1, 2005, and December 31, 2012, are included in this retrospective cohort. Prescriptions were tracked during a follow-up session to gauge PPI use.	In the analysis that controlled for several clinical factors, the use of proton pump inhibitors was linked to incident CKD (hazard ratio (HR) 1.18; 95% CI 1.04–1.51) in both those who started using PPI during the follow-up and those who took it during the initial visit (HR 1.37; 95% CI 1.25–1.50). High PPI dosages raised the incidence of incident CKD (HR 1.92; 95%CI 1.00–6.19) for both people who used high doses for the duration of the follow-up and for any kind of PPI exposure (HR 2.40; 95%CI 1.65–3.46). Following three months of PPI use, there was an increase in the incidence of incident CKD (HR 1.78; 95% CI 1.39–2.25) between the third and sixth months and (HR 1.30; 95% CI 1.07–1.72) following the sixth month.	PPI use has been linked to an increased incidence of CKD incidents. After three months of exposure, this relationship becomes noticeable and is stronger for high dosages.
Laville, et al. 2018 [20]	Western Europe	Europe (France)	Thirty-three outpatients with CKD (eGFR between 15 and 60 ml min-1.73 m-2) are part of the CKD-REIN cohort. At study enrollment, we looked at the daily dosages of pharmacological medicines administered. In relation to kidney function, we evaluated the frequency and contributing factors of incorrect drug prescriptions (i.e., contraindications or excessive dosages) in patients with CKD receiving nephrology care. We also evaluated the effect of the GFR estimation equation on the prevalence estimations.	At least one incorrect medicine prescription had been given to half of the patients. Medications for cardiovascular disease, gout, and diabetes made up the majority of prescriptions that were improperly written. Different GFR equations produced different percentages of improper prescriptions: 52% with the CKD-EPI equation, 47% with the de-indexed CKD-EPI equation, and 41% with the CG equation. The results of a multiple logistic regression analysis indicated that patients who were male (1.28 [1.07; 1.53]), had diabetes (1.34 [1.06; 1.70]), had a high BMI (1.58 [1.25; 1.99]), and had a low GFR (10.2 [6.02; 17.3]) had significantly higher odds ratios [95% confidence intervals] for inappropriate prescriptions. The number of pharmaceuticals per patient increased the chance of receiving at least one incorrect prescription (P for trend < 0.0001). Consequently, the odds ratio for patients who received at least 11 prescribed prescriptions was 5.88 [4.17; 8.28] compared to those who received fewer than 5.	Our findings highlight the difficulty in managing medications for individuals with chronic kidney disease (CKD), for whom improper prescriptions seem to be widespread.
Giusti, et al. 2021 [21]	West-South Central Region of the US	United States	This study examines the relationship between the rate of decrease in renal function and chronic PPI use among veterans with CKD (G3a–G4).	Dialysis, all-cause death, and the progression of CKD were all markedly more likely to occur in the PPI group (aHR, 1.83; 95% CI, 1.53 to 2.19; aHR, 1.84; 95% CI, 1.26 to 2.67; and aHR, 1.34; 95% CI, 1.08 to 1.65, respectively). Although the difference was not statistically significant, the PPI cohort also showed a trend toward the development of metabolic acidosis (aHR, 1.34; 95% CI, 0.998 to 1.80).	According to the research, long-term PPI use increases the risk of renal disease development and is linked to a higher death rate in CKD patients.
Lazarus, et al. 2016 [22]	North America	United States of America	The purpose of this prospective community-based cohort study is to measure the incidence of CKD and PPI use.	The use of proton pump inhibitors was linked to incident chronic kidney disease (CKD) in three different analyses: unadjusted (hazard ratio [HR], 1.45; 95% CI, 1.11-1.90); adjusted (HR, 1.50; 95% CI, 1.14-1.96); and adjusted (adjusted HR, 1.35; 95% CI, 1.17-1.55) with PPI ever use modeled as a time-varying variable.	The use of PPIs increases the risk of AKI and CKD independently.
Antoniou, et al. 2015 [23]	Region of the Americas	Ontario, Canada	This population-based cohort study aims to evaluate older patients' risk of acute kidney injury and acute interstitial nephritis.	Acute interstitial nephritis (0.32 vs. 0.11 per 1000 person-years; HR 3.00, 95% CI 1.47 to 6.14) and acute kidney damage (13.49 vs. 5.46 per 1000 person-years; HR 2.52, 95% CI 2.27 to 2.79) were more common in people who were given PPI than in people who were not.	When older patients began PPI medication, they were more likely to develop interstitial nephritis and acute renal injury.
Pannoi, et al. 2022 [24]	Southeast Asia	Thailand	This is a retrospective cohort to evaluate the hypomagnesemia and chronic kidney disease (CKD) hazards related to PPI usage.	There was a statistically significant correlation between PPIs and CKD (adjusted hazard ratio [HR] = 3.753, 95% CI = 2.385–5.905).	In this hospital-based population, PPIs linked to CKD had a statistically significant effect.
Zhang, et al. 2023 [25]	North America	United States of America	The purpose of this multicenter prospective matched cohort study is to evaluate the relationship between the use of PPIs and the risk of acute renal injury during hospitalization.	PPI use and the risk of post-hospitalization AKI were not statistically significantly associated after adjusting for baseline co-morbidities, drug use histories, and demographic characteristics (rate ratio [RR], 0.91; 95% CI, 0.38 to 1.45). According to baseline AKI status, there were no significant associations between the use of PPIs and the incidence or risk of recurrent AKI (RR, 1.01; 95% CI, 0.27 to 1.76) at all.	Regardless of the individuals' baseline AKI status, PPI usage following the index hospitalization was not a significant risk factor for post-hospitalization AKI and the advancement of renal disorders.
Svanström, et al. 2018 [26]	European Region	Denmark	This prospective cohort study uses proton pump inhibitor-treated rheumatoid arthritis patients to evaluate their risk of acute kidney injury.	Acute kidney damage risk was considerably higher in patients who used proton pump inhibitors (hazard ratio 2.30, 95% confidence interval 1.26–4.20). A significantly higher risk of the secondary outcome of any major renal incident was also linked to the use of proton pump inhibitors (hazard ratio 2.61, 95% confidence interval 1.80–3.80).	In this group study of rheumatoid arthritis patients, using a proton pump inhibitor was linked to a much higher rate of acute renal injury.
Scott, et al. 2019 [27]	Region of the Americas	United States of America	To assess the risk of acute kidney damage (AKI) in HIV patients taking proton pump inhibitors (PPI), a cohort study was carried out.	21,643 patients in all—6,000 PPI and 15,643 non-PPI—met all research requirements. When compared to controls, the PPI cohort had a twice as high incidence of AKI (2.12, hazard ratio: 1.46-3.1).	A nationwide cohort study confirmed an elevated incidence of AKI in patients taking PPIs.
Grant, et al. 2019 [28]	European Region	United Kingdom	To find out if PPI use is linked to major adverse renal events (MARE) in patients with CKD, retrospective observational cohort research including patients with the disease is being conducted.	In addition to having a lower estimated glomerular filtration rate (eGFR) and greater proteinuria, the PPI group was younger. After multiple factors were taken into account, the use of PPI was linked to the worsening of MARE (hazard ratio 1.13 [95% confidence interval 1.02–1.25], P = 0.021). Significantly higher proteinuria, comorbidities, and decreased eGFR were also linked to the progression to MARE.	In individuals with CKD, PPI usage was linked to the progression to MARE but not death after controlling for variables such as reduced eGFR, proteinuria, and comorbidities that are known to predict worsening renal function.
Xie, et al. 2016 [10]	Region of the Americas	United States of America	To determine the relationship between the use of proton pump inhibitors and the risk of incident CKD and progression to ESRD, a cohort study was carried out.	CKD and eGFR<60 ml/min per 1.73 m2 were more likely to happen in the PPI group compared to the H2 blockers group (HR, 1.22; 95% CI, 1.18 to 1.26; and HR, 1.28; 95% CI, 1.23 to 1.34, respectively). Patients taking PPI also had a higher chance of having their serum creatinine level double (HR, 1.53; 95% CI, 1.42 to 1.65), their eGFR drop by more than 30% (HR, 1.32; 95% CI, 1.28 to 1.37), and they developing end-stage renal disease (HR, 1.96; 95% CI, 1.21 to 3.18). Additionally, compared to individuals exposed for ≤30 days, we found a graded connection between the length of PPI exposure and the risk of renal outcomes for those exposed for 31–90, 91–180, 181–360, and 361–720 days.	PPI exposure raises the risk of incident CKD, CKD progression, and end-stage renal disease (ESRD).
Pakkir, et al. 2023 [29]	Eastern Mediterranean Region	UAE	The negative effects of using proton pump inhibitors over an extended period of time are the main topic of this review article.	The Atherosclerosis Risk in Communities (ARIC) study, a population-based cohort study, found that PPI users were more likely to get incident CKD.	PPIs have been linked in several studies to the development of renal illnesses, including end-stage renal disease (ESRD), acute kidney injury (AKI), chronic kidney disease (CKD), and acute interstitial nephritis (AIN).
Ikuta, et al., 2022 [30]	Western Pacific Region	Japan	The purpose of this case series study was to examine the relationship between PPI use and the incidence of AKI, as well as the relationship between exposure to macrolide antibiotics and the IRR of AKI in PPI users. The investigation was carried out utilizing computerized medical data at Kyoto University Hospital.	When comparing the period with PPIs to the one without, the estimated IRR and aIRR were 2.46 and 2.01, respectively. The predicted IRR and aIRR for the period including PPIs and macrolide antibiotics in comparison to those involving PPIs alone were 1.26 and 0.82, respectively.	The use of PPIs was linked in the study to an elevated risk of AKI at any stage. On the other hand, no correlation was found between the concurrent use of PPIs and macrolide antibiotics.
Zhang, et al. 2021 [31]	European Region	United Kingdom	462,421 people in the United Kingdom Biobank were part of the prospective cohort study. Self-reported PPI use was documented using an electronic questionnaire and verified by staff members with training. The medical history was used to identify the incident of CKD.	Over a median follow-up of 8.1 years, 7,031 instances of CKD were reported. According to overlap propensity score weighting analysis, regular PPI users were 37% more likely than non-users to experience a CKD episode.	The regular use of PPIs was linked to an increased risk of chronic kidney disease (CKD), according to this large cohort study.
Wu, et al. 2023 [32]	Western Pacific Region	Taiwan	observational studies assessing the relationship between PPI usage and the risk of chronic kidney disease (CKD)	Ten observational studies totaling 6,829,905 people were included. PPI usage was substantially linked to a higher risk of chronic kidney disease (CKD) compared to non-PPI use (RR 1.72, 95% CI: 1.02–2.87, p = 0.03).	PPI use is associated with a higher risk of chronic kidney disease (CKD).
Chen, et al. 2022 [33]	Western Pacific Region	Taiwan	A retrospective cohort study looked at patients with chronic kidney disease (CKD) who were followed up on after starting acid-suppressant drugs (H2RA and PPI). The goal was to find out how these drugs affected renal and survival outcomes.	For the cohort study, individuals who were new to using PPI, H2RA, or neither (as controls) were taken into account. Users of H2RA and PPI showed adjusted hazard ratios of 1.15 (0.91–1.45) for ESRD and 1.83 (1.65–2.03) for death, and 0.40 (95% confidence interval, 0.30–0.53) for ESRD and 0.64 (0.57–0.72) for death, respectively.	While PPI usage was linked to an increased risk of overall mortality in patients with CKD but not ESRD, dose-dependent H2RA use was linked to a decreased risk of both ESRD and overall mortality in CKD patients.
Wakabayashi, et al. 2021 [34]	Western Pacific Region	Japan	A retrospective observational study was conducted to evaluate the relationship between senior hypertension patients' declining kidney function and a modest dose of proton pump inhibitors.	The 152 patients in the study had a mean age of 74.5 years and were 57.9% male. PPI users made up 35.5% of the group (low dose: 17.1%; high dose: 18.4%). In the high-dose PPI group, the eGFR was considerably lower (P = 0.009) than in the low-dose PPI or non-user groups. Similarly, in the low-dose PPI and non-user groups, there were no significant changes in Scr between baseline and three years prior to treatment; but, in the high-dose PPI group, there was a significant rise in Scr (P = 0.0009).	Low PPI dosages may be secure in therapeutic situations.
Ikuta, et al. 2021 [35]	Western Pacific Region	Japan	In this nested case-control study, the researchers wanted to find out if taking proton pump inhibitors (PPIs) along with antibiotics (penicillins, macrolides, cephalosporins, or fluoroquinolones) or non-steroidal anti-inflammatory drugs (NSAIDs) raised the risk of acute kidney injury (AKI).	317 cases of AKI were found over a mean follow-up of 2.4 (SD, 1.7) years (incidence rate: 6.1/10 000 person-years). Compared to prior PPI usage, the use of PPIs is currently linked to a greater risk of AKI (unadjusted OR, 4.09; 95% CI, 3.09 to 5.44). In comparison to the present use of PPIs alone, the unadjusted ORs of AKI for the combination of PPIs and NSAIDs, cephalosporins, and fluoroquinolones were 3.92 (95% CI, 2.40 to 6.52), 2.57 (1.43 to 4.62), and 3.08 (1.50 to 6.38), respectively.	In the modified model, the effects of co-using PPIs with NSAIDs, cephalosporins, or fluoroquinolones are still considerable. The studies on the absolute risk of AKI validated the results from the nested case-control research.
Peng, et al. 2016 [36]	Western Pacific Region	Taiwan	Proton pump inhibitor (PPI) use may be linked to acute renal damage and nephritis, according to a case-control study. Research is required to determine whether PPI use increases the risk of renal function decline in patients with renal disorders.	In individuals with renal illness, the use of PPIs was linked to a notably increased risk of ESRD (adjusted OR = 1.88, 95% CI = 1.71–2.06). The adjusted OR of all PPI types together was 1.92 (95% CI = 1.74–2.13) for individuals with cumulative DDD <100, and 1.74-fold (95% CI = 1.52–2.00) for those with cumulative DDD ≥ 100.	The use of PPIs raises a patient's risk of end-stage renal disease (ESRD). Patients diagnosed with renal disease must have their PPI prescriptions appropriately coordinated with continuous monitoring of their renal function.
Nadri, et al. 2014 [37]	Eastern Mediterranean Region	UAE	Omeprazol-induced acute granulomatous interstitial nephritis (GIN)	With stable CKD stage IV, omeprazole-induced GIN led to enough renal recovery, enabling dialysis independence.	With a score of 6, the Naranjo adverse medication response likelihood scale indicated that omeprazole was most likely the cause.
Li, et al. 2020 [38]	Western Pacific Region	China.	a multicenter retrospective cohort study assessing the association between the use of proton pump inhibitors (PPI) and the risk of hospital-acquired acute kidney injury (HA-AKI) in pediatric patients admitted to hospitals	37,296 (27.2%), 1,760 (4.2%), and 3,514 (8.3%) of the 42,232 children analyzed used PPI, histamine 2 receptor antagonists (H2RA), and HA-AKI while in the hospital. Over 85 percent of PPI prescriptions were issued with the intention of preventing gastro-duodenal lesions in children. When compared to people who didn't use PPI (OR = 1.37; 95% CI = 1.23–1.53) or who used H2RA (OR = 1.24; 95% CI = 1.01–1.52), people who did use PPI were much more likely to have HA-AKI. For children of various ages, genders, PPI subtypes, and administration styles, the associations were constant. There was a higher impact on children with chronic kidney illness (OR, 3.37; 95% CI, 2.46–4.62) and those in critical care (OR, 1.54; 95% CI, 1.33–1.78). The risk of HA-AKI increased even when PPI was used within the recommended dosage range.	PPIs were widely used and associated with an increased risk of HA-AKI in hospitalized children in China.
Sampathkumar, et al. 2013 [39]	South-East Asia Region	India	There are four cases total—one male and three female. Two PPIs—pantoprazole and one each of omeprazole and esomeprazole—were implicated. After receiving medication for an average of four weeks, AIN appeared.	PPIs affect kidney function.	In India, PPI-induced AIN is probably not well known or treated. It has non-specific symptoms. If the doctor has strong suspicions about this illness, they should cease the medication, do a renal biopsy if necessary, and begin steroid therapy to stop the kidney disease from getting worse.

**Table 2:** CASP checklist to assess the quality of studies The asterisk sign (*) indicates ‘yes.’

Questions	Hart, et al. 2019 [14]	Guedes, et al. 2020 [15]	Aurora, et al. 2016 [16]	Lu, et al. 2022 [17]	Hung, et al. 2018 [18]	Rodríguez-Poncelas, et al. 2018 [19]	Laville, et al. 2018 [20]
Did the review address a focused question?	*	*	*	*	*	*	*
Did the authors look for the correct types of papers?	*	*	*	*	*	*	*
Do you think all the essential, relevant studies were included?	*	*	*	*	*	*	*
Did the review’s authors do enough to assess the quality of the included studies?	*	*	*	*	*	*	*
If the review results have been combined, was it reasonable to do so?	*	*	*	*	*	*	*
What are the overall results of the review?	*	*	*		*	*	*
How precise are the results?	*	*	*	*	*	*	*
Can the results be applied to the local population?	*		*	*	*	*	
Were all important outcomes considered?	*	*	*	*	*	*	*
Are the benefits worth the harm and costs?	*	*	*	*	*	*	*
Results	Good	Fair	Good	Fair	Good	Good	Fair

**Table 3:** CASP checklist to assess the quality of studies The asterisk sign (*) indicates ‘yes.’

Cont.	Giusti, et al. 2021 [21]	Lazarus, et al. 2016 [22]	Antoniou, et al. 2015 [23]	Pannoi, et al. 2022 [24]	Zhang,. et al. 2023 [25]	Svanström, et al. 2018 [26]	Sutton, et al. 2019 [27]	Grant, et al. 2019 [28]
Did the review address a focused question?	*	*	*	*	*	*	*	*
Did the authors look for the correct types of papers?	*	*	*	*	*	*	*	*
Do you think all the essential, relevant studies were included?		*	*	*	*	*	*	*
Did the review’s authors do enough to assess the quality of the included studies?	*	*	*	*	*	*	*	*
If the review results have been combined, was it reasonable to do so?	*	*	*	*	*	*	*	*
What are the overall results of the review?	*	*		*	*	*	*	*
How precise are the results?	*	*	*	*	*	*	*	*
Can the results be applied to the local population?	*	*	*	*	*		*	*
Were all important outcomes considered?	*	*	*	*	*	*	*	*
Are the benefits worth the harm and costs?	*	*	*	*	*	*	*	*
Results	Fair	Good	Fair	Good	Good	Fair	Good	Good

**Table 4:** CASP checklist to assess the quality of studies The asterisk sign (*) indicates ‘yes.’

Cont.	Xie, et al. 2016 [10]	Maideen, et al. 2023 [29]	Ikuta, et al. 2022 [30]	Zhang, et al. 2021 [31]	Wu, et al. 2023 [32]	Chen, et al. 2022 [33]	Wakabayashi, et al. 2021 [34]
Did the review address a focused question?	*	*	*	*	*	*	*
Did the authors look for the correct types of papers?	*	*	*	*	*	*	*
Do you think all the essential, relevant studies were included?	*	*	*	*	*	*	*
Did the review’s authors do enough to assess the quality of the included studies?	*	*	*	*	*	*	*
If the review results have been combined, was it reasonable to do so?	*	*	*	*	*	*	*
What are the overall results of the review?	*	*	*	*	*	*	*
How precise are the results?	*	*	*	*	*	*	*
Can the results be applied to the local population?	*	*	*	*	*	*	*
Were all important outcomes considered?	*	*	*	*	*	*	*
Are the benefits worth the harm and costs?	*	*	*	*	*	*	*
Results	Good	Fair	Good	Good	Good	Good	Good

**Table 5:** CASP checklist to assess the quality of studies The asterisk sign (*) indicates ‘yes.’

Cont.	Ikuta, et al. 2021 [35]	Peng, et al. 2016 [36]	Nadri, et al. 2014 [37]	Li, et al. 2020 [38]	Sampathkumar, et al. 2013 [39]
Did the review address a focused question?	*	*	*	*	*
Did the authors look for the correct types of papers?	*	*	*	*	*
Do you think all the essential, relevant studies were included?	*	*	*	*	*
Did the review’s authors do enough to assess the quality of the included studies?	*	*	*	*	*
If the review results have been combined, was it reasonable to do so?	*	*	*	*	
What are the overall results of the review?	*		*	*	*
How precise are the results?	*	*	*	*	
Can the results be applied to the local population?	*	*	*	*	*
Were all important outcomes considered?	*	*	*	*	*
Are the benefits worth the harm and costs?		*	*	*	*
Results	Fair	Fair	Good	Good	Fair

Discussion

**Figure 2:**
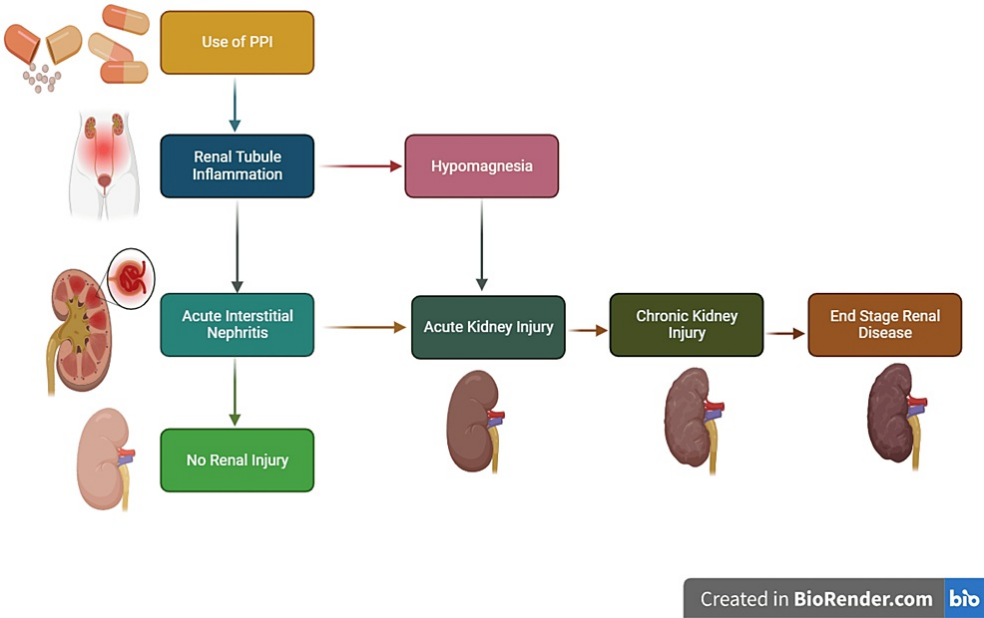
Pathophysiology of PPI and CKD proggression Relationship between PPI use and the development of CKD PPIs - proton pump inhibitors, CKD - chronic kidney disease

PPIs are associated with the development of CKD (Figure 2). PPIs have been generated to prevent the stomach from secreting stomach acids and raise the pH of the stomach fluid. They distinguish themselves from other medications employed for the treatment of gastrointestinal illnesses by additionally blocking the final phase in the formation of hydrochloric acid, which prevents the enzyme called H+/K+-ATPase from carrying out its function and preventing the substitution of K+ for hydrogen ions. PPIs are currently the medicine of preference since this mechanism increases their effectiveness of resistance [40,41]. PPIs obstruct the activity of the enzyme by interacting with its binding partner and linking it covalently to irrevocable inhibitory residues of cysteine. The proton pump cannot regenerate after the process has begun, and acid generation only happens after the creation of freshly generated enzymes. Irreversible suppression guarantees that the drug remains effective for a period of between 24 and 48 hours [42-44].

Among the drugs that are most frequently prescribed in the United States is PPI, and anywhere from 25% to 70% of scripts are thought to be written without the proper justification [5]. The amount of time used regularly exceeds advised limits [45]. Additionally, there is a tendency for PPI usage in young children and babies [46]. In 2013, more than fifteen million individuals took prescription PPIs, spending over $10 billion on them in America [47]. According to 29 investigations, 70% of those medications are unneeded, and 25% of chronic PPI consumers may stop taking their medication without incurring any side effects [48].

PPIs are classified as weak acidic compounds that vary primarily in their particles and have a similar fundamental molecular framework as other weak acids. Whenever delivered, they have no effect; however, if the environment is acidic, they produce sulfamide metabolites or sulfenic acid. PPIs have a gastro-resistant layer to stop the medicine from activating and degrading earlier than it reaches the intended location. After management, the medications are promptly assimilated and active due to their approximately one- to two-hour plasma half-lives. PPIs undergo metabolism by hepatic enzymes called cytochrome P450, which therefore might impact how other drugs are biotransformed. Additionally, alterations in the pH of the stomach may modify how different medicines are absorbed [49,50].

But as time passed, PPIs started to be indiscriminately administered to individuals for purposes that weren't intended, for a more significant duration than was advised, and by people who were taking their medication [51]. Aside from the side effects listed above, more and more evidence is showing that PPIs can cause bone fractures, respiratory infections, magnesium deficiency, dementia, and kidney diseases like acute kidney injury (AKI), acute interstitial nephritis (AIN), and CKD [52,53].

AIN is one of the infrequent side effects that PPIs are most frequently linked to. The interstitium and the tubules of the kidneys are both involved in this immune-driven response. Infection, blood abnormalities, autoimmune diseases, and medications all have the potential to cause it. Tubule tissue cells are initially damaged, and then a mononuclear inflammatory infiltration with a predominance of T cells is seen. The renal cortex may start to scar as a result of the infiltration spreading, which will also cause a decline in kidney function. Individuals with drug-induced AIN can acquire CKD with fibrosis of the interstitial space and atrophy of the tubular walls if no improvement in symptoms is apparent after stopping the alleged medication and starting steroids [54,55].

According to estimates, AIN accounts for 8% of cases of acute kidney damage, between 70% and 90% of which are drug related. Antibiotics, PPIs, and NSAIDs constitute the primary pharmacological categories associated with AIN [56,57]. Numerous studies published after the initial case study from 1992 [39,58] confirmed the link between AIN and PPI consumption. PPI users had a three-fold increased incidence of AIN, according to Antoniou et al. (95% CI 1.47-6.14; n = 290,592). It is not known what causes the inflammation of the kidneys in these people, but the buildup of PPIs and/or their related metabolites in the interstitial tubes and the autoimmune reaction that follows have been suggested as a possible explanation [23,59].

Acute kidney injury could be accelerated by the rapid decline in kidney functioning caused by tubulointerstitial pathologies. The investigation into the underlying causes of this condition led to the discovery of AIN, which a kidney biopsy frequently confirms. Gallium-67 scintigraphy is a technique that can be used to distinguish between AIN and acute necrosis of the tubes in cases where biopsy is not advised. About 30% of those who improve from AKI continue to have an elevated risk of developing CKD [60-63]. A further adverse consequence of PPI usage is hypomagnesemia. PPIs were linked to a twice-rise in the probability of having low magnesium levels, according to research of 9,818 participants (95% CI: 1.36 to 2.93). The physiological process underlying the reduction in magnesium, or Mg, levels caused by PPIs is not fully understood. Reduced urine concentrations imply that the intestinal tract is where magnesium depletion occurs. Studies suggest that CKD and low blood levels of magnesium (0.7 mmol/L) are related. Prolonged interstitial nephritis can eventually result in failure of the kidneys and, in extreme situations, CKD [56,64].

Lazarus et al. were the initial researchers to propose a link between PPIs and CKD in 2016. The authors investigated the possibility that histamine H2-receptor antagonists (H2) and PPIs individually pose a possibility for CKD. The researchers employed 10,482 participants from the Atherosclerosis Risk in Communities (AIRC) research group. 10,482 individuals in ARIC had an average follow-up of 13.9 years. 248,751 people in the confirmation population were monitored for an average duration of 6.2 years. The investigation was then conducted again with 248,751 Geisinger Health System patients. The results were comparable across each of the groups of individuals, and PPI usage was linked to an increased likelihood and a 1.17 to 1.5-fold greater chance of developing CKD. The relationship had to be verified using PPIs. In ARIC, there were a total of 56 occurrences of CKD events in the group of 322 baseline PPI users (14.2 per 1,000 person-years) and 1,382 events among the 10,160 baseline those who were not users (10.7 per 1,000 person-years), compared to non-users who had a greater beginning BMI and were taking antihypertensive, pain reliever, or statin drug medications. Among 322 initial PPI users, the anticipated 10-year overall likelihood of developing CKD was 11.8%; however, their predicted risk would have been 8.5% had patients avoided using PPIs (absolute risk difference, 3.3%) [65].

A work by Xie et al. linked PPIs with CKD and the development of failure of the kidneys. The participants selected were tracked for a period of five years (n = 173,321 for PPIs and n = 20,270 for H2RA). According to the investigation, people taking PPIs had a 1.28 times increased chance of getting CKD, along with a 1.96 times higher risk of going on to develop chronic renal failure. As in other investigations, no connection between H2RA and renal illness was discovered [10]. A three-week treatment with PPIs, such as omeprazole, lansoprazole, and pantoprazole at a dose of thirty milligrams per kilogram in rodents merely resulted in an increase in serum levels of IS, according to the study. The increased amounts of liver CYP2E1 protein, which promotes IS production, are likely responsible for this impact. This process may help to explain the link between the use of PPIs and a higher risk of developing CKD [17]. We should follow specific guidelines while prescribing PPIs [66].

## Conclusions

Our systematic review of the impact of PPIs on kidney function and CKD progression has provided valuable insights into this important medical topic. Through a comprehensive analysis of existing research, it has become evident that the use of PPIs is associated with certain risks to kidney health. While PPIs have been effective in managing various gastrointestinal conditions, their potential adverse effects on kidney function cannot be ignored. The findings of this review suggest that healthcare professionals should exercise caution when prescribing PPIs, especially for patients with pre-existing kidney conditions or those at risk of developing CKD. Close monitoring of kidney function and thoughtful consideration of alternative treatment options should be integral parts of clinical decision-making. In summary, while PPIs have revolutionized the management of acid-related disorders, their impact on kidney function underscores the importance of a balanced approach to their use, taking into account individual patient factors and a commitment to minimizing potential harm to kidney health.
